# Editorial: Relationship between intestinal microbiome and vasculitis

**DOI:** 10.3389/fcimb.2023.1165730

**Published:** 2023-04-04

**Authors:** Wenzhu Song, Shengxiao Zhang, Xiaofeng Li, Chong Gao, Jun Cai, Yafeng Li

**Affiliations:** ^1^ School of Public Health, Shanxi Medical University, Taiyuan, Shanxi, China; ^2^ Department of Rheumatology, The Second Hospital of Shanxi Medical University, Taiyuan, Shanxi, China; ^3^ Shanxi Provincial Key Laboratory of Rheumatism Immune Microecology, Taiyuan, Shanxi, China; ^4^ Key Laboratory of Cellular Physiology at Shanxi Medical University, Ministry of Education, Taiyuan, China; ^5^ Brigham and Women’s Hospital, Harvard Medical School, Boston, MA, United States; ^6^ Fuwai Hospital, Chinese Academy of Medical Sciences and Peking Union Medical College, Beijing, China; ^7^ Department of Nephrology, Shanxi Provincial People’s Hospital (Fifth Hospital) of Shanxi Medical University, Taiyuan, China; ^8^ Core Laboratory, Shanxi Provincial People's Hospital (Fifth Hospital) of Shanxi Medical University, Taiyuan, China; ^9^ Shanxi Provincial Key Laboratory of Kidney Disease, Taiyuan, China; ^10^ Academy of Microbial Ecology, Shanxi Medical University, Taiyuan, China

**Keywords:** intestinal microbiome, immunoregulation, autoimmunity, vasculitis, dysbiosis

The gastrointestinal tract harbors a complex and dynamic microbiome, i.e., intestinal microbiome. Accumulating evidence suggests that gut microbiota is involved in the pathogenesis of numerous intestinal and systemic autoimmune diseases. Commensal flora facilitates the regulation of the maturation of the mucosal immune system and constitutes an important protective barrier to gut integrity (Shi et al., 2017), helping prevent the microbiota of healthy individuals from triggering adaptive immune responses (Manfredo Vieira et al., 2018). Dynamic interactions between the gut microbiota and the host immune system are critical for intestinal homeostasis and inflammatory suppression. According to previous studies, altered intestinal flora composition, namely, dysbiosis, is featured in connective tissue diseases and primary vasculitis.

Accordingly, we held such a topic as “Relationship Between Intestinal Microbiome and Vasculitis”, in which we aimed to clarify the relationship between microbiome and lymphocyte subsets, explore mechanisms of immunologic imbalance caused by the microbiome in autoimmune diseases like vasculitis, and seek for potential therapeutic approaches targeting the gut microbiota for vasculitis. In this topic, five out of six articles were finally accepted, with 31 contributing authors. Of note, these five papers primarily focused on two major diseases: vasculitis and cardiovascular diseases (CVDs).

## Intestinal microbiome and vasculitis

Although the relationship between intestinal microbiome and some immune diseases has been investigated widely (Levy et al., 2017; Shi et al., 2017; Zhou B. et al., 2020), it remains under-addressed in terms of the occurrence and development of vasculitis. As a response, one article by Hu et al. investigated the landscape of IgAN, Kawasaki disease, and IgA vasculitis, showing intestinal microbiome dysbiosis in these diseases. This paper’s contribution lies in its discovery of some shared bacteria that may be closely associated with IgA deposition, which could offer a new scientific theory for the pathogenesis of the three diseases. As a complementary paper, a review by Sun et al. summarized the relationship between intestinal microbiome and different types of vasculitis, which concern small-, medium-, large-, and variable-vessel vasculitis in terms of biological mechanisms. Its contributions relate to its detailed explanation of some potential immune mechanisms, such as impaired intestinal permeability caused by an abnormal immune response, and abnormal microbial metabolites such as SCFAs and H_2_S. As far as we are concerned, these papers made a clear explanation of intestinal microbiome and vasculitis, and should gain the attention of scholars in this field.

Another study by Hong et al. focused on autistic children with and without atopic dermatitis (AD), which analyzed the diversity, compositions, and functional pathways among the two groups, and concluded more prominent susceptibility to gastrointestinal disorders in AD individuals. The key point it addressed represents the exploration of significant differences in gut microbiota and urinary organic acids between the two groups, suggesting the importance of gut microbiota in the development of autistic children with AD.

## Intestinal microbiome and cardiovascular diseases

In recent years, numerous studies demonstrated the important role of microbial communities in the development of CVDs (Witkowski et al., 2020; Zhou W. et al., 2020). Undoubtedly, viruses represent an integral part of microbiome. An article by Tan et al. aimed to explore the causal associations between cytomegalovirus (CMV) and nine CVDs, showing increased risk of coronary artery disease, peripheral arterial disease, and deep vein thrombosis by a genetic predisposition to anti-CMV IgG levels. Its contribution lies in its first direct evidence that anti-CMV IgG levels are genetically associated with some CVDs by using Mendelian randomization (MR). With single-nucleotide polymorphisms (SNPs) strongly associated with exposure as instrumental variables (Boehm and Zhou, 2022), MR allows for causal inference between exposure and outcomes, and since the assignment of parent alleles occurs randomly at gametogenesis and it is therefore independent of environmental factors, MR facilitates the reduction of confounding and strengthens the exposure-to-outcome association (Ference et al., 2021), overcoming the limitations of observational epidemiological studies. Personally, as a novel important statistical method, MR holds great clinical prospects and should be promoted to make a more detailed analysis.

In the other paper by Qian et al., the effects of gut microbiota on CVDs were reviewed. It focused on coronary atherosclerosis, hypertension, and heart failure, suggesting an intestinal microbiota dysbiosis. Moreover, it fully explored some gut microbiota metabolites involved in the immune mechanism for the disease development in detail, from bile acid to trimethylamine-N-oxide, and to short-chain fatty acids, contributing to the discovery of novel treatment, such as fecal microbiota transplantation, dietary intervention, use of probiotics, 3,3-dimethylbutanol, and exercise. What it addressed emphasizes its recent findings on the gut microbiota–CVD relationship, and exploration of some known gut microbiota-related metabolites and their relationships with CVD development. We believe that this paper lays a solid foundation for further research on gut microbiota and CVDs and the immune mechanisms involved.

## Discussion and prospect

These five papers concentrated on research hotspots, detecting the relationship between gut microbiota and immune diseases, and mechanistically linking microbiota to disease-related changes in immune cells. Although most of the conclusions were based on cross-sectional studies, this topic helps fill the gap in the relationship between intestinal microbiome and vasculitis.

We believe that this topic could set the scene for experts’ opinion exchanges, speeding up an academic understanding of intestinal microbiome and vasculitis. Hopefully, this topic could spark more exciting and scoping research and discussion that will not only promote better and deeper communication on vasculitis’ pathogenicity mechanisms, but also introduce more intestinal microbiome-related therapeutic approaches for vasculitis.

## Author contributions

WS was responsible for the manuscript writing. SZ helped polish the manuscript. YL, XL, CG, and JC are the topic Editors and offered some constructive comments on this manuscript. All authors contributed to the article and approved the submitted version.
